# Evaluation of a multi-epitope vaccine PME for *Pasteurella multocida* in mouse model

**DOI:** 10.3389/fimmu.2025.1652907

**Published:** 2025-09-01

**Authors:** Rui Zhang, Lu Dai, Yizhen Jia, Haoran Qi, Junfeng He, Jiaxin Cheng, Xuejun Gao, Liancheng Lei, Feng Liu

**Affiliations:** ^1^ College of Animal Science and Technology, Yangtze University, Jingzhou, Hubei, China; ^2^ College of Veterinary Medicine, Jilin University, Changchun, China

**Keywords:** *Pasteurella multocida*, multi-epitope vaccine, DNA vaccine, immunogenicity, cross-protection

## Abstract

*Pasteurella multocida* (*P. multocida*) is the pathogen responsible for swine pasteurellosis, which can impede their growth and even cause death, leading huge economic losses to the global pig industry. *P. multocida* can be divided into 5 serotypes, and existing vaccines have low cross-immunity protection. Therefore, developing a vaccine that can provide effective cross-protection is essential for preventing swine pasteurellosis and reducing the abuse of antibiotics. In this study, six dominant antigenic proteins of *P. multocida* (PlpE, OmpA, OmpH, VacJ, Omp87 and Cp39) were selected. Through bioinformatics methods, 20 B-cell epitopes, 7 CTL epitopes and 11 Th-cell epitopes were predicted. The multi-epitope antigen PME was constructed by connecting these epitopes with linkers, and then the recombinant protein His-PME and the recombinant plasmid pcDNA3.1-PME could effectively stimulate immunized mice to produce antibodies, IL-4 and IFN-γ. The protection rates of His-PME group and pcDNA3.1-PME group were 62.5% and 75% against *P. multocida* serotype A, and 87.5% and 100% against *P. multocida* serotype D, respectively. Furthermore, the pathological lung damages in the His-PME group and the pcDNA3.1-PME group were significantly alleviated, and the bacterial loads in lung tissues were significantly decreased. These results indicated that the subunit vaccine His-PME and the DNA vaccine pcDNA3.1-PME can effectively resist the infection of *P. multocida* and have good immunogenicity and cross-protection. Therefore, the multi-epitope vaccine PME can be regarded as a candidate vaccine for the prevention of *P. multocida* infection.

## 1 Introduction

*Pasteurella multocida* (*P. multocida*) is the causative agent of swine pasteurellosis, with fibrinous pleuropneumonia, pharyngitis, hemorrhagic inflammation, and septicemia as the main clinical features (1). The most acute infection leads to the death of the affected pigs within 12 hours, and the mortality rate of acute infection can reach up to 100% (2). This disease is highly contagious and mainly infects healthy pigs through direct contact, indirect contact and air transmission, causing serious economic losses to the pig industry worldwide (3). *P. multocida* can be classified into five serotypes (A, B, D, E and F) based on capsular antigens, among which serotypes A, B, D and E can cause disease in pigs. The current prevalent serotypes are A and D (4).

At present, the prevention and control of swine pasteurellosis mainly relies on antibiotic therapy and vaccination (5). However, the increasing use of antibiotics has led to drug-resistant strains, posing severe challenges to the control of swine pasteurellosis (6). *P. multocida* vaccines mainly include inactivated vaccines, attenuated vaccines, and subunit vaccines, with inactivated vaccines and attenuated vaccines prevalent in clinical practices (7). Inactivated vaccines have high safety, but only target a single serotype and cannot effectively provide cross-protection (8). Attenuated vaccines, though with the advantages of strong and long-lasting antibody responses, have the risk of reversion to virulence (9).

Subunit vaccines not only can provide better cross-protection but also avoid the risk of reversion to virulence, making it an important research direction for *P. multocida* vaccines (10). Outer membrane protein, as one of the main virulence factors of *P. multocida*, has been widely used in vaccine research and has been proven to induce good immune protection (11). After challenging *P. multocida* serotype A, the protection rates of outer membrane lipoprotein (PlpE) when immunizing mice with different adjuvants ranged from 80% to 100%, outer membrane protein H (OmpH) was 100%, and the outer membrane protein 87 (Omp87) was 83.3% (12–14). The protection rates of VacJ family lipoprotein (VacJ) was 66.7% against *P. multocida* serotype B (15). The outer membrane protein A (OmpA) expressed in prokaryotic cells was found to be immunogenic by immunoblot analysis (16). The immunization of chickens with natural adhesion protein Cp39 resulted in a 100% protection rate against *P. multocida* strain P-1059 (serotype A) (17). However, due to the complex structure of natural antigenic protein, there are few effective epitopes exposed on the surface (18).

As a novel type of vaccine, multi-epitope vaccines predict the B lymphocyte (B-cell) and T lymphocyte (T-cell) epitopes of antigens through bioinformatics methods, and prepare vaccines by concatenating dominant epitopes, which can activate the host’s humoral and cellular immunity (19). Moreover, multi-epitope vaccines have the advantages of high safety and cross-protection (20, 21). DNA vaccines can elicit long-lasting immunity by delivering exogenous genes that encode antigenic proteins into host cells, thereby enabling the stable expression of these antigenic proteins (22). Meanwhile, without intricate protein purification, multi-epitope DNA vaccines are straightforward to develop, and can be engineered through screening to concatenate dominant epitopes, thereby providing efficient immune protection and cross-immune protection (23, 24).

In this study, the B-cell and T-cell epitopes of six antigen proteins of *P. multocida*, namely PlpE, OmpA, OmpH, VacJ, Omp87 and Cp39, were predicted by bioinformatics methods. These epitopes were concatenated through flexible linkers (GSG) to obtain the multi-epitope PME, and the physicochemical properties such as antigenicity index and hydrophilicity were analyzed by bioinformatics. Then, the prokaryotic expression recombinant vector pET30a-PME and the eukaryotic expression recombinant vector pcDNA3.1-PME were constructed to obtain the recombinant protein His-PME and the recombinant plasmid pcDNA3.1-PME. By evaluating the immune efficacy of His-PME and pcDNA3.1-PME on mice, it was shown that the subunit vaccine and DNA vaccine prepared based on the multi-epitope PME had good immune protection against *P. multocida* serotypes A and D.

## 2 Materials and methods

### 2.1 Strains, plasmid and culture conditions

*P. multocida* was cultured in brain and heart infusion (BHI; Solarbio, Beijing, China) containing 10% newborn bovine serum (EVERY GREEN, Hangzhou, China). When cultivating *E. coli* containing pET-30a plasmid, kanamycin (50 μg/mL) was added to Luria-Bertani (LB; Solarbio, Beijing, China). When cultivating *E. coli* containing pcDNA3.1 plasmid, ampicillin (100 μg/mL) was added to LB medium. All strains and plasmids in this experiment were listed in **Table 1**, and the sources of software were listed in **Supplementary Table S1**.

**Table 1:** Bacterial strains and plasmids used in this study.

Strain and plasmid	Description	Source
Pasteurella multocida		
PM-HD17	*Pasteurella multocida* serotype A	Our Laboratory
PM-RH43	*Pasteurella multocida* serotype D	Our Laboratory
E. coli		
BL21	Expression protein for recombinant vector	Takara
DH5α	Recombinant *plasmid* amplification	Takara
Plasmid		
pET-30a-*pme*	pET-30a carrying *pme* gene	Sangon Biotech
pcDNA3.1-*pme*	pcDNA3.1 carrying *pme* gene	Sangon Biotech

### 2.2 Prediction of B-cell and T-cell epitopes

The amino acid sequences of six antigenic proteins of *P. multocida*, namely PlpE, OmpA, OmpH, VacJ, Omp87 and Cp39, were downloaded from the NCBI database. In Immune Epitope Database and Tools (IEDB), Chou & Fasman Beta-Turn Prediction, Emini Surface Accessibility Prediction, Karplus & Schulz Flexibility Prediction, Kolaskar & Tongaonkar Antigenicity, Parker Hydrophilicity Prediction, and Bepipred Linear Epitope Prediction 2.0 were used to predict the B-cell epitopes of the above six proteins (25–30). Linear B-cell epitopes with high surface accessibility, high proportion of β-turns and random coils, high antigenicity index, high hydrophilicity, and strong flexibility were selected as the final dominant B-cell epitope sequences.

Predict cytotoxic T lymphocyte (CTL) epitopes using ANN 4.0, Consensus, netMHCcons, PickPocket, SMMPMBEC in IEDB. Select pig major histocompatibility complex I (MHC I) alleles SLA-1*0401, SLA-2*0401, and SLA-3*0401 as receptors, and screen the CTL epitopes with a length of 9 amino acids and an IC_50_ value less than or equal to 500 (31–36). MHCpred (https://www.ddg-pharmfac.net/mhcpred/MHCPred/) was used to predict helper T lymphocyte (Th-cell) epitopes, with the DRB1*0101 allele as the receptor. Short peptides with an IC_50_ value less than or equal to 500 and a higher logIC_50_ score was selected as Th-cell epitopes (37).

### 2.3 Design and prokaryotic expression of multi-epitope protein PME

The predicted B-cell and T-cell antigen epitopes were concatenated using a flexible linker (GSG) to obtain the multi-epitope protein PME (38). Bioinformatics tools such as Expasy ProtParam, ToxinPred, AllerTOP v2.0, VaxiJen v2.0, SOLpro, DNAstar, SignalP-6.0, and DeepTMHMM were used to analyze the basic physicochemical properties, toxicity, allergic reactions, antigenicity, solubility, hydrophilicity, flexibility, surface accessibility, signal peptides, and transmembrane structure of multi-epitope protein PME (39–41).

Prokaryotic expression assay was performed as described earlier, with some modifications (42–44). The nucleotide sequence of the multi-epitope protein PME was codon optimized, synthesized by Sangon Biotech (Shanghai, China), and connected to the prokaryotic expression vector pET30a. The recombinant plasmid pET30a-PME was transformed into *E. coli* BL21 (DE3), and cultured in LB (200 mL) medium with 50 μg/mL kanamycin. When OD_600_ was 0.6, the culture was induced with 1 mM Isopropyl β-D-1-thiogalactopyranoside (IPTG) at 37°C for 5 h, centrifuged at 8000 rpm, and then ultrasonically treated. The precipitate was collected by centrifugation, purified by Ni-NTA affinity chromatography and dialysis. Then, the obtained protein His-PME was identified by SDS-PAGE and stored at -80°C.

### 2.4 Secondary structure prediction and tertiary structure modeling

The secondary structure of multi-epitope protein PME was predicted using SOPMA, and its immunological potential was evaluated (45). The tertiary structure was modeled using AlphaFold 3, and the conformational rationality and quality was evaluated using PDBsum and ProSA-web (46, 47).

### 2.5 Predicting linear and conformational B-cell epitopes

After completing the tertiary structure modeling of PME through AlphaFold 3, the ElliPro tool in the IEDB database was further used to predict the linear B-cell epitopes and conformational B-cell epitopes in PME (the minimum score was set to the default value of 0.5, and the maximum distance was set to the default value of 6) (48). The higher PI value of the predicted epitopes proved that they were more prominent on the surface of the protein and more recognized and bound by antibodies (49).

### 2.6 Molecular docking and molecular dynamics simulation

Toll-like receptor family (TLR family) is an important class of pattern recognition receptors (PRRs) that activates immune responses by recognizing pathogen-associated molecular patterns (PAMPs) (50). We obtained the structural coordinates of TLR2 (PDB ID: 3A7C), TLR4 (B: 3VQ2), MHC I (PBD ID: 3V52), and major histocompatibility complex II (MHC II; PBD ID: 2P24) in Mus musculus from the Protein Data Bank (https://www.rcsb.org). The ClusPro server (https://cluspro.org/login.php) was utilized for the docking of PME with the four receptors, namely TLR2, TLR4, MHC I and MHC II (51). Visualization analysis of the interaction of the docking structures was conducted using PyMOL software (https://pymol.org), and molecular dynamics simulation was performed using iMODS to evaluate the stability of the docking structures (52).

### 2.7 Immune response simulation

To evaluate the potential immune response of the multi-epitope protein PME, C-lmmSim was used to simulate immune responses (53). The random seed was set to 12345 by default, and the simulation volume and simulation step were set to 10 and 540, respectively. The immune program consisted of three injections, and the time step lengths of the three injections were set to 1, 84, and 252, respectively. Each time step lengths was equal to 8 hours in real life.

### 2.8 Extraction of recombinant plasmid pcDNA3.1-PME

The nucleotide sequence of the multi-epitope protein PME was codon optimized, and connected to the eukaryotic expression vector pcDNA3.1. The recombinant plasmid pcDNA3.1-PME was transformed into *E. coli* DH5α for overnight culture, and then transferred to LB medium (2.4 L) containing 100 μg/mL ampicillin at a ratio of 1:100 and cultured for 16 h. After centrifugation at 8000 rpm for 10 min, the plasmid was extracted using EndoFree Plasmid MaxiPrep Kit (HLINGENE, Shanghai, China) and stored at -80°C.

### 2.9 Immunoblotting analysis of His-PME protein

The purified protein His-PME was electrophoresed on 12% SDS-PAGE gel and then transferred onto the PVDF membrane. The membrane was incubated with anti-His antibody (1:10000; Solarbio, Beijing, China) overnight at 4°C. After being washed with TBST for 3 times, each for 5 min, the membrane was incubated with goat anti-mouse IgG/Alkaline Phosphatase (1:10000; Beyotime, Shanghai, China) at room temperature for 2 h.

### 2.10 Immunization and challenge in mice

Six-week-old female SPF BALB/c mice (18-20 g) were purchased from Experimental Animal Center of the Three Gorges University, and the detailed immunization information was shown in **Table 2**. All animal experiments were approved by the Animal Ethics Committee of the Yangtze University. In this study, His-PME, pcDNA3.1-PME, inactivated *P. multocida* (serotypes A and D), pcDNA3.1, and PBS were mixed with GEL 01 RP adjuvant (V/V 10:1), and inoculated into mice on days 1, 14 and 28, respectively. Among them, the groups (8 mice/group) of pcDNA3.1-PME and pcDNA3.1 were inoculated into the tibialis anterior muscle of the hind limbs of mice, while the others were inoculated into the subcutaneous tissue of the back of mice. On the 35th day, mice were intraperitoneally challenged with *P. multocida*. The 7-day survival status, clinical symptoms, and clinical scores of these groups were observed and recorded. The scoring was as follows: health (0 point), lethargy (1 point), emaciation (2 points), trembling and weakness of limbs (3 points), hind limb paralysis (4 points), death (5 points) (54).

**Table 2:** Immunization and challenge dose information in mice.

Group	Immunization	Dose of immunization	Dose of challenge
His-PME-A	His-PME+GEL 01 RP	250 μg/per mouse	*P. multocida* serotype A (1.05×10^2^CFU)
Adjuvant-A	PBS+GEL 01 RP	0.1 mL/per mouse	*P. multocida* serotype A (1.05×10^2^CFU)
pcDNA3.1-PME-A	pcDNA3.1-PME+GEL 01 RP	200 μg/per mouse	*P. multocida* serotype A (1.05×10^2^CFU)
pcDNA3.1-A	pcDNA3.1+GEL 01 RP	200 μg/per mouse	*P. multocida* serotype A (1.05×10^2^CFU)
Inactivated *P. multocida*-A	inactivated *P. multocida* serotype A+GEL 01 RP	1.03×10^8^CFU/per mouse	*P. multocida* serotype A (1.05×10^2^CFU)
PBS-A	PBS	0.1 mL/per mouse	*P. multocida* serotype A (1.05×10^2^CFU)
His-PME-D	His-PME+GEL 01 RP	250 μg/per mouse	*P. multocida* serotype D (1.04×10^7^CFU)
Adjuvant-D	PBS+GEL 01 RP	0.1 mL/per mouse	*P. multocida* serotype D (1.04×10^7^CFU)
pcDNA3.1-PME-D	pcDNA3.1-PME+GEL 01 RP	200 μg/per mouse	*P. multocida* serotype D (1.04×10^7^CFU)
pcDNA3.1-D	pcDNA3.1+GEL 01 RP	200 μg/per mouse	*P. multocida* serotype D (1.04×10^7^CFU)
Inactivated *P. multocida*-D	inactivated *P. multocida* serotype D+GEL 01 RP	3.9×10^8^CFU/first immunization/per mouse 3.6×10^8^CFU/second immunization/per mouse 3.5×10^8^CFU/third immunization/per mouse	*P. multocida* serotype D (1.04×10^7^CFU)
PBS-D	PBS	0.1 mL/per mouse	*P. multocida* serotype D (1.04×10^7^CFU)

### 2.11 Lung bacterial load and histopathological analysis

The mice were subjected to the attack protocol as described above. Since mice began to die sequentially after 8 hours, we elected to euthanize them at the 8-hour time point. The lungs (0.1-0.15g) of the mice were aseptically collected and homogenized using tissue disruptor (NewZongKe, Wuhan, China). Then, these samples were serially diluted with 0.9% NaCl, plated on BHI plates containing 10% newborn bovine serum, and incubated at 37°C for 24 h for colony counting. Meanwhile, the partial lung tissue of the mice was fixed in 10% formalin for histo-pathological analysis. The scoring of pathological changes was as follows: no lesions (0 point), mild pathological changes (1 point), moderate pathological changes (2 points), severe pathological changes (3 points), and extremely severe pathological changes (4 points) (55). The evaluation contents included hemolysis, inflammatory cell infiltration, hemorrhage, and widening of the alveolar septa.

### 2.12 The serum antibody levels of mice

After immunization, serum samples were collected from the tail vein of each group of mice on days 0, 13, 27, and 34, respectively, and the antibody levels were detected by indirect ELISA (56). 100 μL of the fragmented *P. multocida* serotype A or D (100 μg/mL) was added to each well and coated overnight at 4°C. After washing with PBST, 150 μL of 2% BSA was added to each well and incubated at 37°C for 2 h. Serum samples from each group (1:400) were added as primary antibodies to the wells and incubated at 37°C for 1 h. Goat anti-mouse IgG HRP (1:5000; Beyotime, Shanghai, China) was added as a secondary antibody to the wells and incubated at 37°C for 1 h. Finally, TMB buffer (Solarbio, Beijing, China) was added and incubated at room temperature for 5 min, and then the OD_450_ was measured.

### 2.13 Cytokine Analysis

Serum samples were collected from the mice in each group 34 days after immunization, and the levels of interferon gamma (IFN-γ) and interleukin-4 (IL-4) cytokines in the serum were detected using ELISA kit (YUANJU, Shanghai, China). The absorbance was measured at 450 nm.

### 2.14 Statistical analysis

All statistical analyses were performed using the unpaired Student’s *t* test by GraphPad Prism (version 9.0; GraphPad, La Jolla, CA), and *P < 0.05 was considered statistically significant.

## 3 Results

### 3.1 Prediction of B-cell and T-cell epitopes

The average score of B-cell epitopes of PlpE, OmpA, OmpH, VacJ, Omp87 and Cp39 was calculated by Chou & Fasman Beta-Turn Prediction, Emini Surface Accessibility Prediction, Karplus & Schz Flexibility Prediction, Kolaskar & Tongaonkar Antigenicity, and Parker Hydrophilicity Prediction (**Table 3**). The epitopes with scores higher than the average score and that marked with “E” in the Bepipred Linear Epitope Prediction 2.0 were selected as candidate B-cell epitopes (**Table 4**). The CTL and Th-cell epitopes of the above six proteins were predicted using ANN4.0, Consensus, netMHCcons, PickPocket, NetMHCpan4.1EL, SMMPMBEC, and MHCpred. Epitopes with IC_50_≤500 and higher logIC_50_ scores were selected as candidate Th-cell epitopes (**Table 5**). Epitopes with IC_50_ ≤ 500 and that included in NetMHCpan 4.1 EL were selected as candidate CTL epitopes (**Table 5**). In this study, 20 B-cell epitopes, 7 CTL epitopes and 11 Th-cell epitopes were screened (**Table 6**).

**Table 3:** The average scores of B-cell epitopes obtained by different prediction methods.

Annotation	Chou & Fasman Beta-Turn	Emini Surface Accessibility	Karplus & Schulz Flexibility	Kolaskar & Tongaonkar Antigenicity	Parker Hydrophilicity
PlpE	1.062741	1.000036	1.019851	1.000971	2.708971
OmpA	0.982662	1.000009	0.991188	1.037858	1.761442
OmpH	0.983354	1.000003	0.996015	1.025982	1.800578
VacJ	1.000025	0.999996	1.002406	1.026167	1.234033
Omp87	1.009436	1.00001	1.004628	1.01712	1.752366
Cp39	0.984254	1.000009	0.999919	1.021527	2.025879

**Table 4:** Scoring and labeling of B-cell epitopes of six proteins.

Annotation	Epitopes	Chou & Fasman Beta-Turn	Emini Surface Accessibility	Karplus & Schulz Flexibility	Kolaskar & Tongaonkar Antigenicity	Parker Hydrophilicity	Bepipred Linear Epitope 2.0
PlpE	SEPSSAP	1.247	1.009	1.085	1.011	4.8	E
	SQQSSFK	1.123	1.253	1.108	1.012	4	E
	QPSADYK	1.171	1.901	1.024	1.016	4.357	E
OmpA	VRSDYKV	0.999	1.459	1.048	1.087	2.443	E
	DYKVYDK	1.103	3.92	0.997	1.042	3.414	E
	YKVYDKE	1	4.065	1.008	1.04	3.1	E
	VYDKEPA	1.004	2.027	1.057	1.046	3.157	E
	THSTQVS	1.03	1.262	1.034	1.049	3.971	E
	HSTQVSP	1.11	1.352	1.034	1.071	3.529	E
	VDYRPDI	1.071	1.328	1.012	1.052	1.814	E
	NKCDSVK	1.166	1.104	1.044	1.044	4.657	E
OmpH	AGYSQKY	1.131	1.918	1.034	1.031	3.171	E
	GYSQKYV	1.109	1.409	1.043	1.077	2.343	E
	YSQKYVK	1.03	2.848	1.038	1.085	2.343	E
	SQKYVKQ	1.007	3.148	1.02	1.064	3.471	E
	DYAQSKV	1.026	1.533	1.025	1.062	3.529	E
VacJ	SYSPPLR	1.226	1.838	1.019	1.062	1.471	E
	QSQDPYI	1.14	1.928	1.071	1.041	2.957	E
	KVSTPKQ	1.059	2.601	1.062	1.035	3.929	E
Omp87	HYNSVGR	1.156	1.054	1.005	1.026	2.843	E
	YNSVGRY	1.183	1.213	1.01	1.034	2.271	E
	QAFSSSK	1.077	1.162	1.048	1.019	3.443	E
	YPLDREH	1.05	2.455	1.014	1.024	2.157	E
	NVPDYSD	1.296	1.723	1.032	1.018	4.286	E
	VPDYSDP	1.29	1.657	1.033	1.059	3.586	E
	YSDPSRV	1.204	1.684	1.058	1.053	3.386	E
	DPSRVRA	1.067	1.587	1.034	1.019	3.629	E
	KPLKKYQ	1.037	4.412	1.042	1.04	2.014	E
	PLKKYQG	1.116	2.183	1.042	1.032	2.014	E
Cp39	GLSDYTY	1.183	1.022	1.002	1.033	2.057	E
	VEQNPPA	1.069	1.365	1.081	1.031	3.343	E

**Table 5:** Screening results of CTL-cell epitopes and Th-cell epitopes.

Annotation	Epitopes	CTL/Th	ANN 4.0 IC50 (nM)	Consensus IC50 (nM)	netMHCcons IC50 (nM)	PickPocket IC50 (nM)	NetMHCpan 4.1 EL	SMMPMBEC IC50 (nM)	MHCpred IC50 (nM)	MHCpred logIC_50_ (M)
PlpE	DVNRVGSEY	CTL	74.11	74.11	232.24	446.909	Yes	39.30339	/	/
	FIYSVLSDV	Th	/	/	/	/	/	/	21.23	7.673
	YIYAIKPDA	Th	/	/	/	/	/	/	96.16	7.017
OmpA	AVELGYDDF	CTL	307.18	307.18	208.42	104.847263969548	Yes	3.19469939860313	/	/
	GIYGEIAQL	Th	/	/	/	/	/	/	27.48	7.561
	DIGSVTAGL	Th	/	/	/	/	/	/	79.07	7.102
	FMPELALRV	Th	/	/	/	/	/	/	100.69	6.997
OmpH	GDDVGVSDY	CTL	41.93	41.93	251.87	359.94793166265	Yes	14.1719626798289	/	/
	AINFKSAEF	Th	/	/	/	/	/	/	490.1	5.309
VacJ	LLEQSQDPY	CTL	105.95	105.95	221.2	456.685289375156	Yes	42.7986430764227	/	/
	TMWDFNYKV	Th	/	/	/	/	/	/	23.99	7.62
	VMLPLYGPA	Th	/	/	/	/	/	/	31.33	7.504
Omp87	QTDAWWKLF	CTL	114.61	114.61	83.54	76.6102662600341	Yes	48.0208673508292	/	/
	STTAFAAPF	CTL	192.12	192.12	126.02	405.441642325188	Yes	262.077658531362	/	/
	YLDRGYAQF	Th	/	/	/	/	/	/	29.85	7.525
	GSDQVDVIY	Th	/	/	/	/	/	/	399.5	6.046
Cp39	GDDVGLSDY	CTL	34.15	34.15	239.9	363.863633791522	Yes	14.2701980321056	/	/
	FAYEGLGTL	Th	/	/	/	/	/	/	43.55	7.361

**Table 6:** B, CTL, and Th-cell epitopes selected from six proteins.

Annotation	Position	B-cell epitopes	Position	CTL-cell epitopes	Position	Th-cell epitopes
PlpE	46-52 81-87 182-188	SEPSSAP SQQSSFK QPSADYK	228-236	DVNRVGSEY	221-229 194-202	FIYSVLSDV YIYAIKPDA
OmpA	136-148 156-163 200-206 323-329	VRSDYKVYDKEPA THSTQVSP VDYRPDI NKCDSVK	77-85	AVELGYDDF	256-264 205-213 173-181	GIYGEIAQL DIGSVTAGL FMPELALRV
OmpH	204-213 252-258	AGYSQKYVKQ DYAQSKV	126-134	GDDVGVSDY	151-159	AINFKSAEF
VacJ	121-127 205-211 229-235	SYSPPLR QSQDPYI KVSTPKQ	202-210	LLEQSQDPY	40-48 149-157	TMWDFNYKV VMLPLYGPA
Omp87	137-144 182-189 610-616 737-744 740-749 769-777	HYNSVGRY QAFSSSK YPLDREH NVPDYSDP YSDPSRVRA KPLKKYQG	13-21 197-205	STTAFAAPF QTDAWWKLF	225-233 403-411	YLDRGYAQF GSDQVDVIY
Cp39	138-144 223-229	GLSDYTY VEQNPPA	134-142	GDDVGLSDY	117-125	FAYEGLGTL

### 3.2 Bioinformatics analysis of PME

**Figure 1:**
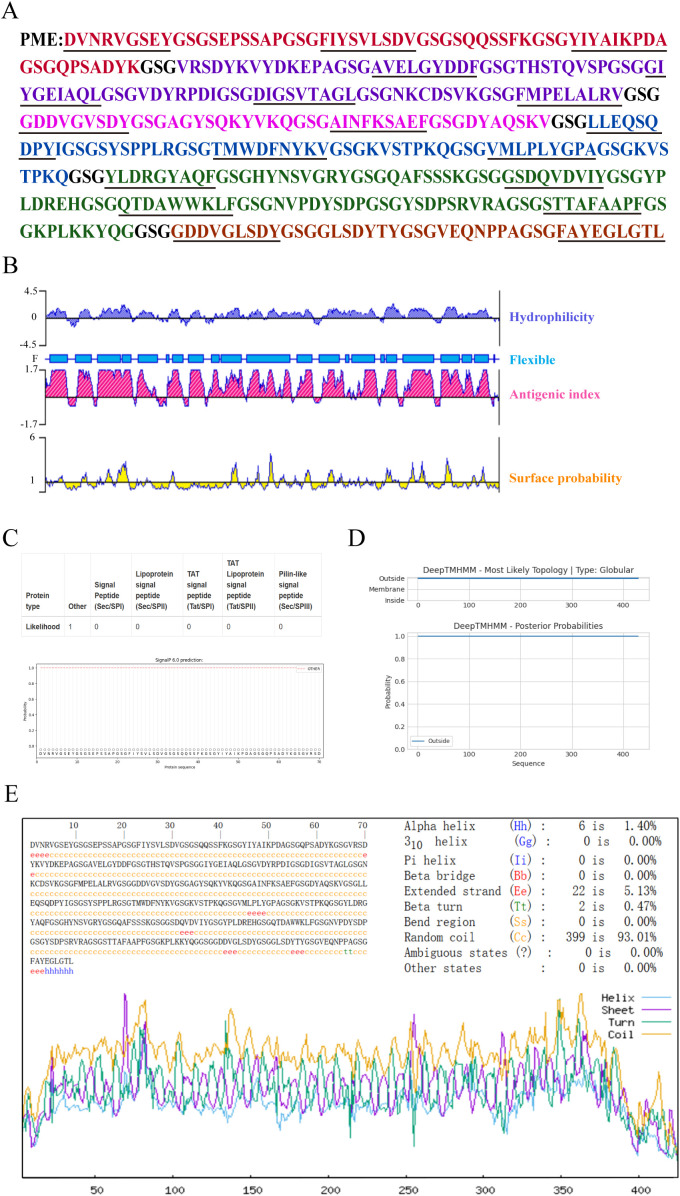
The sequence structure and characteristics of PME. **(A)** The amino acid sequence of PME is presented, with the epitope sequences of six proteins displayed in various colors. The PlpE epitope sequence is highlighted in red, the OmpA epitope sequence in purple, the OmpH epitope sequence in pink, the VacJ epitope sequence in blue, the Omp87 epitope sequence in green, the Cp39 epitope sequence in brown, and the GSG linkers in black. The T-cell epitopes are underlined. **(B)** The hydrophilicity, antigenic index, surface probability, and flexibility assessment of PME. **(C)** The analysis of the signal peptide in PME. **(D)** The prediction of the transmembrane region of PME. **(E)** The secondary structure analysis of PME.

The predicted B-cell and T-cell epitopes were linked by flexible linkers (GSG) to obtain the multi-epitope antigen PME (**Figure 1A**). The prediction results of DNAstar showed that PME had high hydrophilicity, flexibility, and surface accessibility (**Figure 1B**). SignalP-6.0 and DeepTMHMM analysis showed that PME had no signal peptides and transmembrane regions (**Figures 1C, D**). The secondary structure of PME was predicted by SOPMA, and showing that PME was composed of α-helix (1.4%), chain (5.13%), β-turn (0.47%), and random coil (93.01%) (**Figure 1E**).

The isoelectric point (PI), instability index and hydrophilicity of PME were analyzed by ExPaSy ProtParam, and the results showed that the theoretical PI was 4.86, the instability index was 25.48, and the average hydrophilicity (GRAVY) was -0.544. These results indicated that PME carried negative charges and was not prone to degradation, making it a hydrophilic protein. ToxinPred and AllerTOP v2.0 were respectively used to predict the toxicity and allergenicity of PME, and the results indicated that PME was non-toxic and non-allergenic. Using VaxiJen v2.0 to predict the antigenicity of PME, the result was 1.0523, indicating the strong antigenicity of PME. The solubility probability of PME was predicted to be 0.987453 by SOLpro, showing the high solubility of PME (**Table 7**). In conclusion, these results indicated that PME has the characteristics of strong antigenicity, high solubility, negative charge, difficult degradation, with no transmembrane structure, toxicity, and allergenicity.

**Table 7:** The physicochemical properties of the multi-epitope protein PME structure were evaluated.

Item	Result
Theoretical isoelectric point	4.86
Instability index	25.48
Grand average of hydropathicity (GRAVY)	-0.544
Antigenicity	1.0523
Solubility	0.987453
Toxicity	No
Anaphylaxis	No

### 3.3 Analysis of tertiary structure of PME

**Figure 2:**
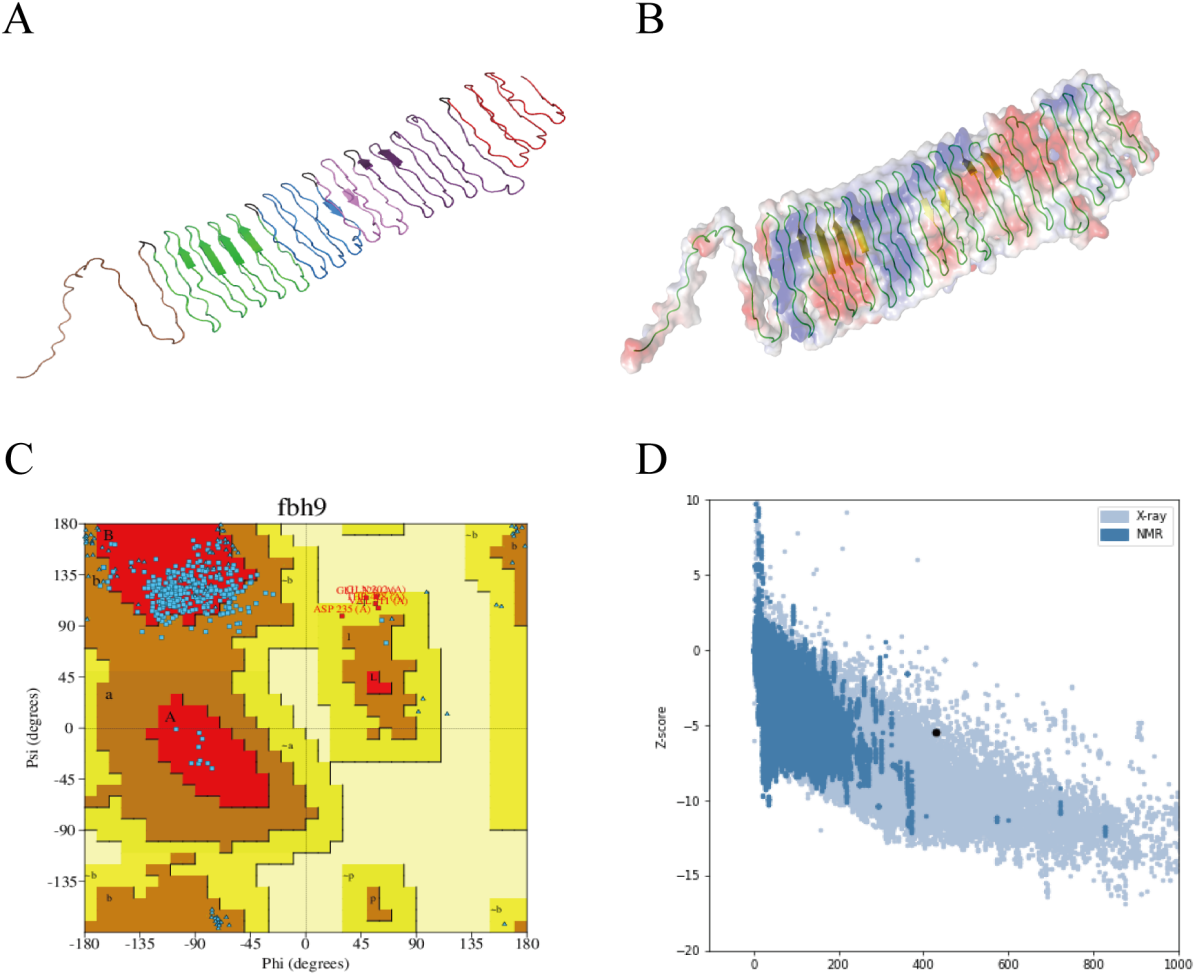
Tertiary structure model and model validation of PME. **(A)** The tertiary structure model of PME. Red represents the epitope sequence of PlpE, purple represents the epitope sequence of OmpA, pink represents the epitope sequence of OmpH, blue represents the epitope sequence of VacJ, green represents the epitope sequence of Omp87, brown represents the epitope sequence of Cp39, and black represents the linkers. **(B)** PyMOL visualizes the secondary structure and surface potential distribution of the PME model. The green lines represent the coiled structure, while the yellow arrows represent the sheet. **(C)** Ramachandran plot of PME. 83.9% of amino acids are in the favored region, and no amino acids are in the disallowed region. **(D)** The Z-score (-5.5) of the PME model.

The tertiary structure of PME was modeled using AlphaFold 3 (**Figure 2A**), and visualized using PyMOL (**Figure 2B**). Ramachandran plot analysis of PME using the PDBsum server showed that 83.9% of the amino acids were in the favorable region, 14.5% were in the allowed region, 1.6% were in the outlier region, and no amino acids were in the disallowed region, indicating that the conformation of the multi-epitope protein PME was reasonable (**Figure 2C**). ProSA-web verified the tertiary structure of PME, with a z-score of -5.5, indicating that PME had a superior 3D protein model (**Figure 2D**). The above results indicated that the multi-epitope protein PME had rationality and excellent quality.

### 3.4 Prediction of linear and conformational B-cell epitopes of PME

**Figure 3:**
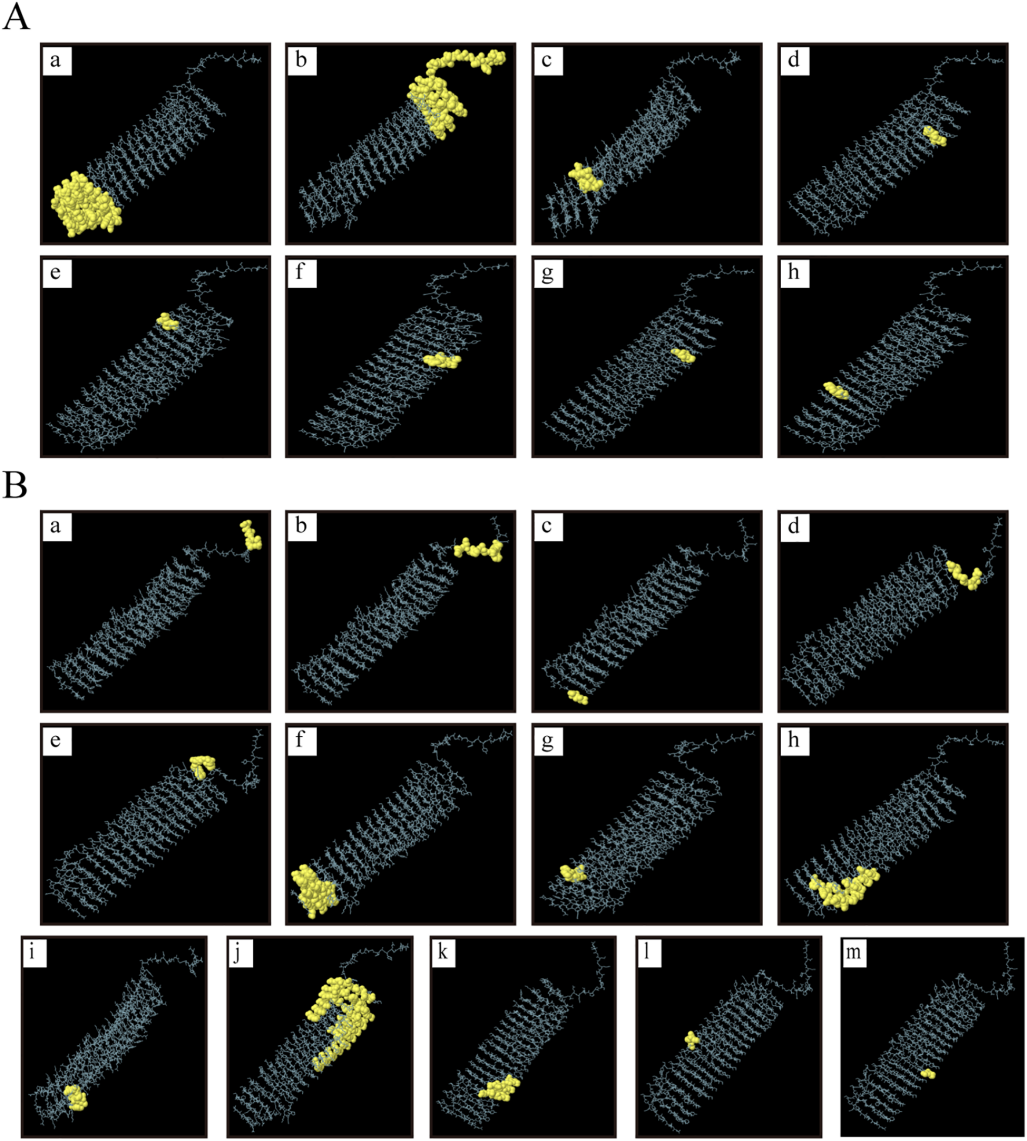
Linear and conformational B-cell epitopes in PME. **(A)** Linear B-cell epitopes are represented by yellow spheres. A total of 8 epitopes (labeled a–h) were identified, and the size of the epitope residues ranges from 5 to 84. **(B)** Conformational B-cell epitopes are represented by yellow spheres. A total of 13 epitopes (labeled a–m) were identified, and the size of the epitope residues ranges from 3 to 89.

The tertiary structure PDB file of multi-epitope protein PME was obtained using AlphaFold 3, and then 8 linear B-cell epitopes (**Figure 3A**) and 13 conformational B-cell epitopes (**Figure 3B**) of PME were predicted by ElliPro in IEDB. The size of the linear epitope ranged from 5 to 84 residues, and their scores ranged from 0.5 to 0.767 (**Table 8**). The size of the conformational B-cell epitope ranged from 3 to 89 residues, and their scores ranged from 0.524 to 0.988 (**Table 9**). These results showed that there were many prominent epitopes on the surface of multi-epitope protein PME, which were easily recognized and bound by antibodies, thus triggering immune response.

**Table 8:** B-cell linear epitope screening results.

Number	Position	Peptide	Number of residues	Score
a	1-84	DVNRVGSEYGSGSEPSSAPGSGFIYSVL SDVGSGSQQSSFKGSGYIYAIKPDAGSG QPSADYKGSGVRSDYKVYDKEPAGSGAV	84	0.767
b	348-429	SDPGSGYSDPSRVRAGSGSTTAFAAPF GSGKPLKKYQGGSGGDDVGLSDYGSG GLSDYTYGSGVEQNPPAGSGFAYEGLGTL	82	0.757
c	88-96	YDDFGSGTH	9	0.61
d	327-334	HGSGQTDA	8	0.583
e	340-344	GSGNV	5	0.559
f	283-289	QFGSGHY	7	0.518
g	305-311	KGSGGSD	7	0.518
h	112-118	AQLGSGV	7	0.5

**Table 9:** Screening results of B-cell conformational epitope.

Number	Position	Peptide	Number of residues	Score
a	A:E424, A:G425, A:L426, A:G427, A:T428, A:L429	EGLGTL	6	0.988
b	A:Q413, A:N414, A:P415, A:P416, A:A417, A:G418, A:S419, A:G420, A:F421, A:A422, A:Y423	QNPPAGSGFAY	11	0.97
c	A:D1, A:V2, A:N3	DVN	3	0.937
d	A:T406, A:Y407, A:G408, A:S409, A:G410, A:V411, A:E412	TYGSGVE	7	0.916
e	A:Y397, A:G398, A:S399, A:G400, A:G401, A:L402, A:S403, A:D404	YGSGGLSD	8	0.842
f	A:V5, A:G6, A:S7, A:E8, A:Y9, A:G10, A:S11, A:G12, A:S13, A:E14, A:P15, A:S16, A:S17, A:A18, A:P19, A:G20, A:S21, A:G22, A:F23, A:I24, A:Y25, A:S26, A:V27, A:L28, A:S29, A:D30, A:V31, A:G32, A:S33, A:G34, A:S35, A:Q36, A:G42, A:S43, A:G44, A:Y45, A:I46, A:Y47, A:A48, A:I49	VGSEYGSGSEPSSAPGSGFIYSVLSDVGSGSQGSGYIYAI	40	0.831
g	A:Y62, A:K63, A:G64, A:S65, A:G66, A:V67	YKGSGV	6	0.765
h	A:Q37, A:S38, A:S39, A:F40, A:K50, A:P51, A:D52, A:A53, A:G54, A:S55, A:G56, A:Q57, A:P58, A:S59, A:K76, A:E77, A:P78, A:A79, A:G80, A:S81, A:G82, A:A83, A:V84, A:G103, A:S104, A:G105, A:G106, A:G125, A:S126, A:G127, A:D128, A:I129, A:G147, A:S148, A:G149	QSSFKPDAGSGQPSKEPAGSGAVGSGGGSGDIGSG	35	0.627
i	A:R68, A:S69, A:D70, A:K72, A:V73, A:Y74	RSDKVY	6	0.612
j	A:G196, A:S197, A:G198, A:G219, A:S220, A:G221, A:G241, A:S242, A:G243, A:G263, A:S264, A:G265, A:S274, A:G275, A:G285, A:S286, A:G287, A:H288, A:Y289, A:N290, A:G296, A:S297, A:G298, A:K305, A:G306, A:S307, A:G308, A:G309, A:D311, A:G318, A:S319, A:G320, A:H327, A:G328, A:S329, A:G330, A:Q331, A:T332, A:D333, A:A334, A:G340, A:S341, A:G342, A:N343, A:V344, A:S348, A:D349, A:P350, A:G351, A:S352, A:G353, A:Y354, A:S355, A:D356, A:P357, A:S358, A:R361, A:A362, A:G363, A:S364, A:G365, A:S366, A:T367, A:T368, A:A369, A:F370, A:A371, A:A372, A:P373, A:F374, A:G375, A:S376, A:G377, A:K378, A:P379, A:L380, A:Q384, A:G385, A:G386, A:S387, A:G388, A:G389, A:D390, A:D391, A:V392, A:G393, A:L394, A:S395, A:D396	GSGGSGGSGGSGSGGSGHYNGSGKGSGGDGSGHGSGQTDAGSGNVSDPGSGYSDPSRAGSGSTTAFAAPFGSGKPLQGGSGGDDVGLSD	89	0.576
k	A:D89, A:D90, A:F91, A:G92, A:S93, A:G94, A:T95, A:H96, A:A112, A:Q113, A:L114, A:G115, A:S116, A:G117, A:V118, A:G137, A:S138, A:G139, A:N140	DDFGSGTHAQLGSGVGSGN	19	0.563
l	A:G171, A:S172, A:G173, A:A174, A:G175	GSGAG	5	0.528
m	A:G159, A:S160, A:G161	GSG	3	0.524

### 3.5 Molecular docking and molecular dynamics simulations

**Figure 4:**
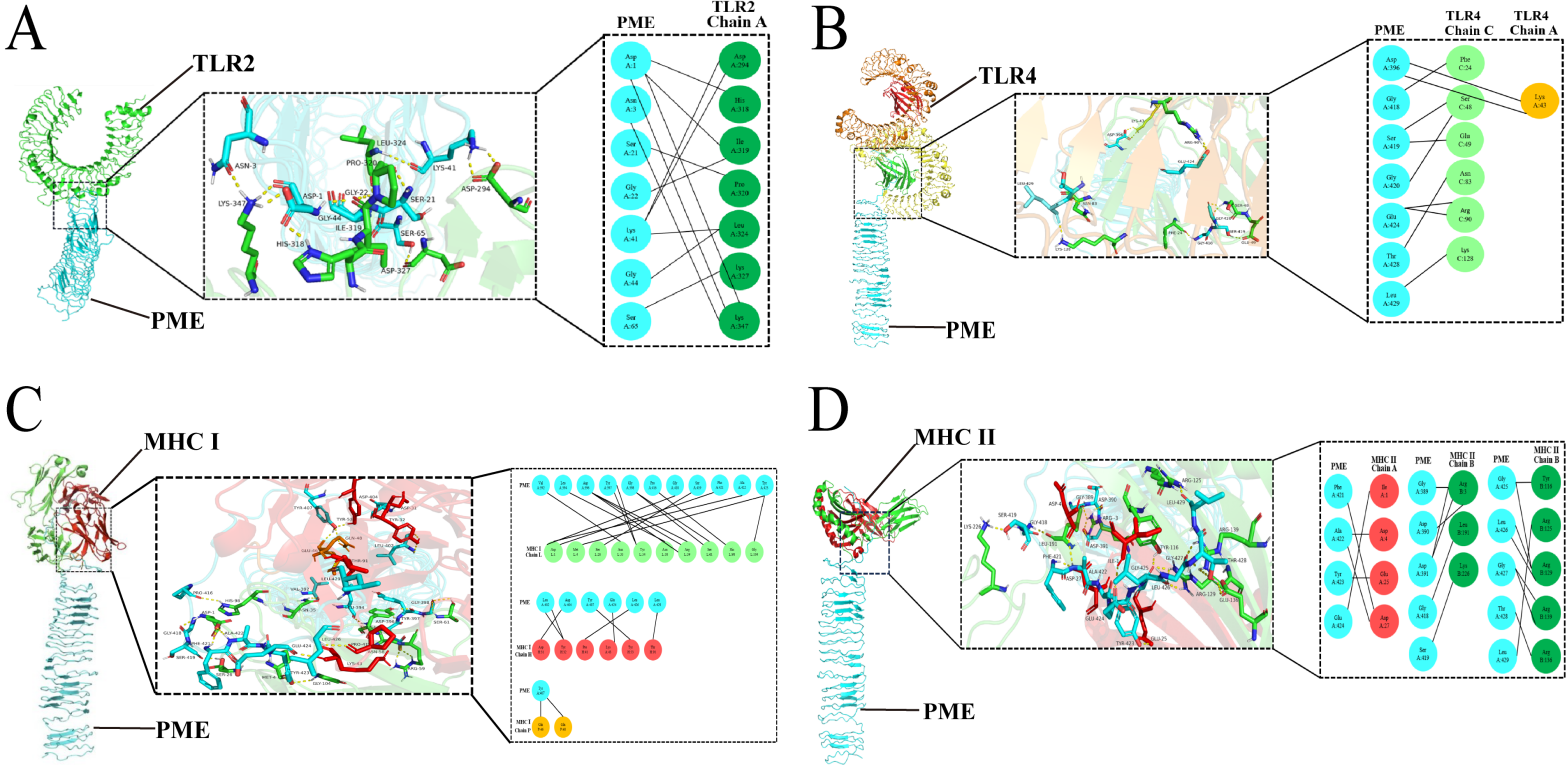
Molecular docking analysis results of PME with TLR2, TLR4, MHC I and MHC II. **(A)** Schematic diagram of molecular docking for PME and TLR2. PME is sky blue, and the A chain of TLR2 is green. **(B)** Schematic diagram of molecular docking for PME and TLR4. PME is sky blue, the A chain of TLR4 is yellow, and the C chain is light green. **(C)** Schematic diagram of molecular docking for PME and MHC I PME is sky blue, the L chain of MHC I is light green, the H chain is red, and the P chain is yellow. **(D)** Schematic diagram of molecular docking for PME and MHC II. PME is sky blue, the A chain of MHC II is red and the B chain is green.

To test the affinity between PME and immune receptors, we used the ClusPro server to dock TLR2, TLR4, MHC I, MHC II with PME, and the lowest energy scores of the docked complexes were -1352.1 (**Figure 4A**), -1373 (**Figure 4B**), -1019.9 (**Figure 4C**), and -964.1 (**Figure 4D**), respectively. The visualization of PME-TR2, PME-TR4, PME-MHC I, and PME-MHC II complexes using PyMOL showed that all these four complexes have multiple hydrogen bond interactions with hydrogen bond distances ranging from 1.7 to 2.8 Å, indicating a high affinity between PME and the four immune receptors (**Table 10**).

**Table 10:** Docking results of protein PME with four receptors.

PME-TLR2	Distance	PME-TLR4	Distance	PME- MHC I	Distance	PME- MHC II	Distance
ASP1- HIS318	2	ASP396- LYS43	1.8 and 1.8	VAL392-ASN35	1.9	Gly389-Arg3	2.2
ASP1-ILE319	2	GLY418-PHE24	1.9	LEU394-ASN58	1.9	Asp390-Arg3	2.1
ASP1-LYS347	1.7 and 1.9	SER419-SER48	1.9	ASP396-ARG59	1.8, 2.2 and 2.2	Asp391-Arg3	1.9 and 2.2
ASN3-LYS347	1.8	SER419-GLU49	2	TYR397-TYR54	1.8 and 2.2	Gly418-Leu191	1.9
SER21-PRO320	1.9	GLY420-SER48	2.1	GLY398-SER61	2.5 and 2.8	Ser419-Lys226	1.7
GLY22-ILE319	2.1	GLU424-ARG90	1.8 and 2.1	LEU402-TYR32	1.8 and 2.5	Phe421-Asp27	2
LYS41-ASP294	1.7 and 1.7	THR428-ASN83	1.9	ASP404- ASP31	2	Ala422-Asp4	2.6
LYS41-LEU324	2.1	LEU429-LYS128	1.7	TYR407-GLU46	1.8	Ala422-Asp27	2.1
GLY44-LEU324	1.8			TYR407-GLN48	2.6	Tyr423-Glu25	2
SER65-ASP327	1.8			TYR407- TYR53	1.8	Glu424-Ile1	2.1
				PRO416-HIS98	2.2	Gly425-Tyr116	1.9
				GLY418-ASP1	2	Gly425-ARG129	1.8
				SER419-SER26	1.9	Leu426-ARG129	1.9 and 2.5
				PHE421-ASP1	2.1	Gly427-Arg139	1.8 and 2.0
						Thr428-Arg136	2.5
				ALA422-ASP1	2.2	Leu429-Arg125	1.8
				TYR423-MET4	1.8		
				TYR423-GLY104	2.2		
				GLU424-LYS43	2		
				LEU426-PRO41	2.2		
				LEU429-THR91	1.9		

**Figure 5:**
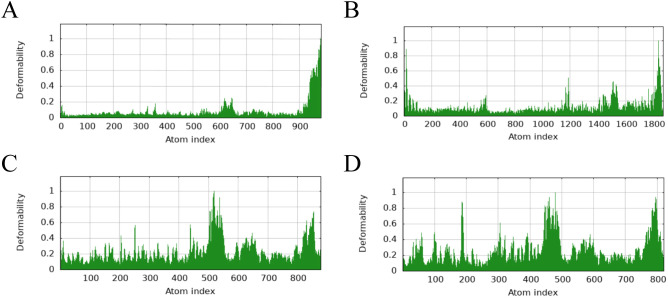
iMODS analysis revealed the main chain deformability of PME-TLR2, PME-TLR4, PME-MHC I, and PME-MHC II. **(A)** The main chain deformability plot of PME-TLR2. **(B)** The main chain deformability plot of PME-TLR4. **(C)** The main chain deformability plot of PME-MHC I. **(D)** The main chain deformability plot of PME-MHC II.

**Figure 6:**
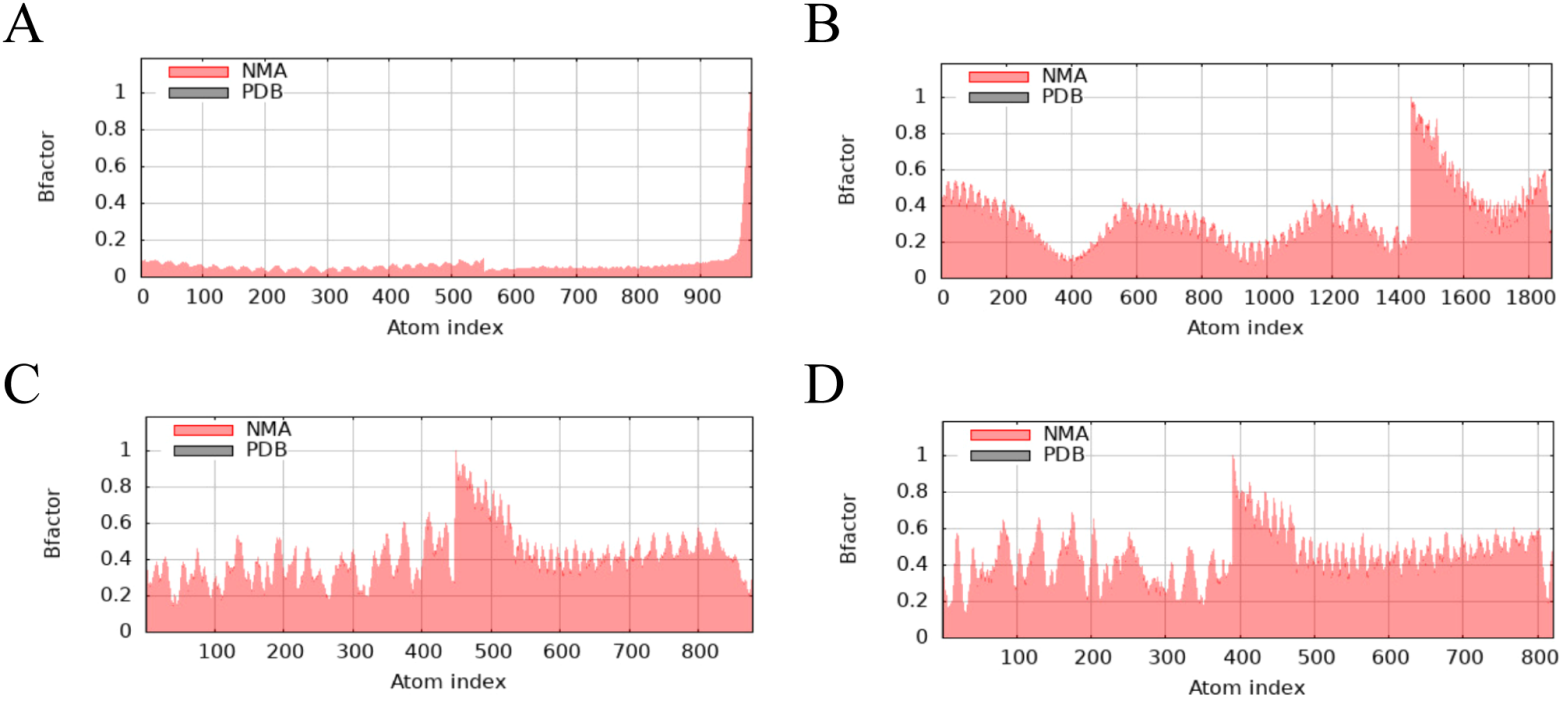
iMODS analysis revealed the B factor of PME-TLR2, PME-TLR4, PME-MHC I, and PME-MHC II. **(A)** The B factor plot of PME-TLR2. **(B)** The B factor plot of PME-TLR4. **(C)** The B factor plot of PME-MHC I. **(D)** The B factor plot of PME-MHC II.

**Figure 7:**
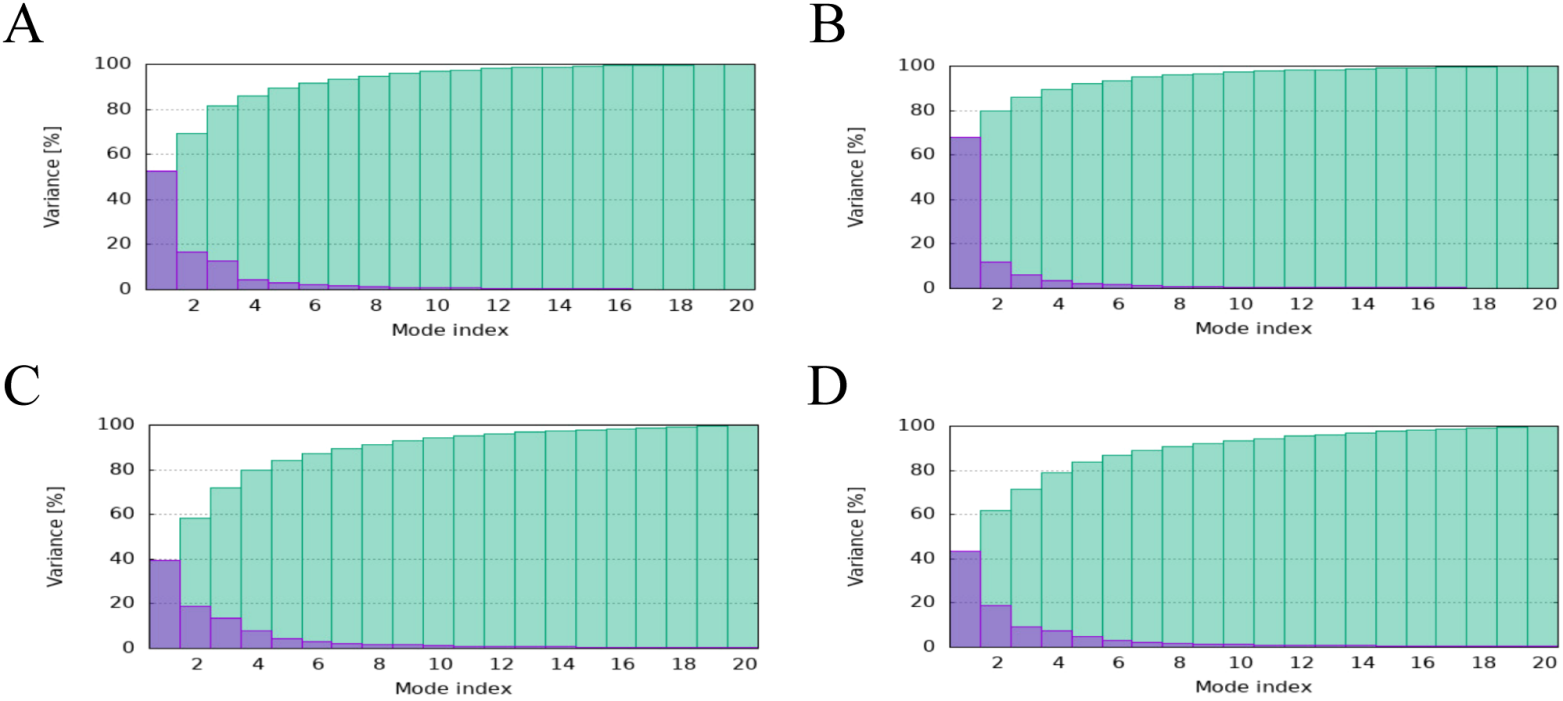
iMODS analysis revealed the Variance of PME-TLR2, PME-TLR4, PME-MHC I, and PME-MHC II. **(A)** The variance plot of PME-TLR2. **(B)** The variance plot of PME-TLR4. **(C)** The variance plot of PME-MHC I. **(D)** The variance plot of PME-MHC II.

**Figure 8:**
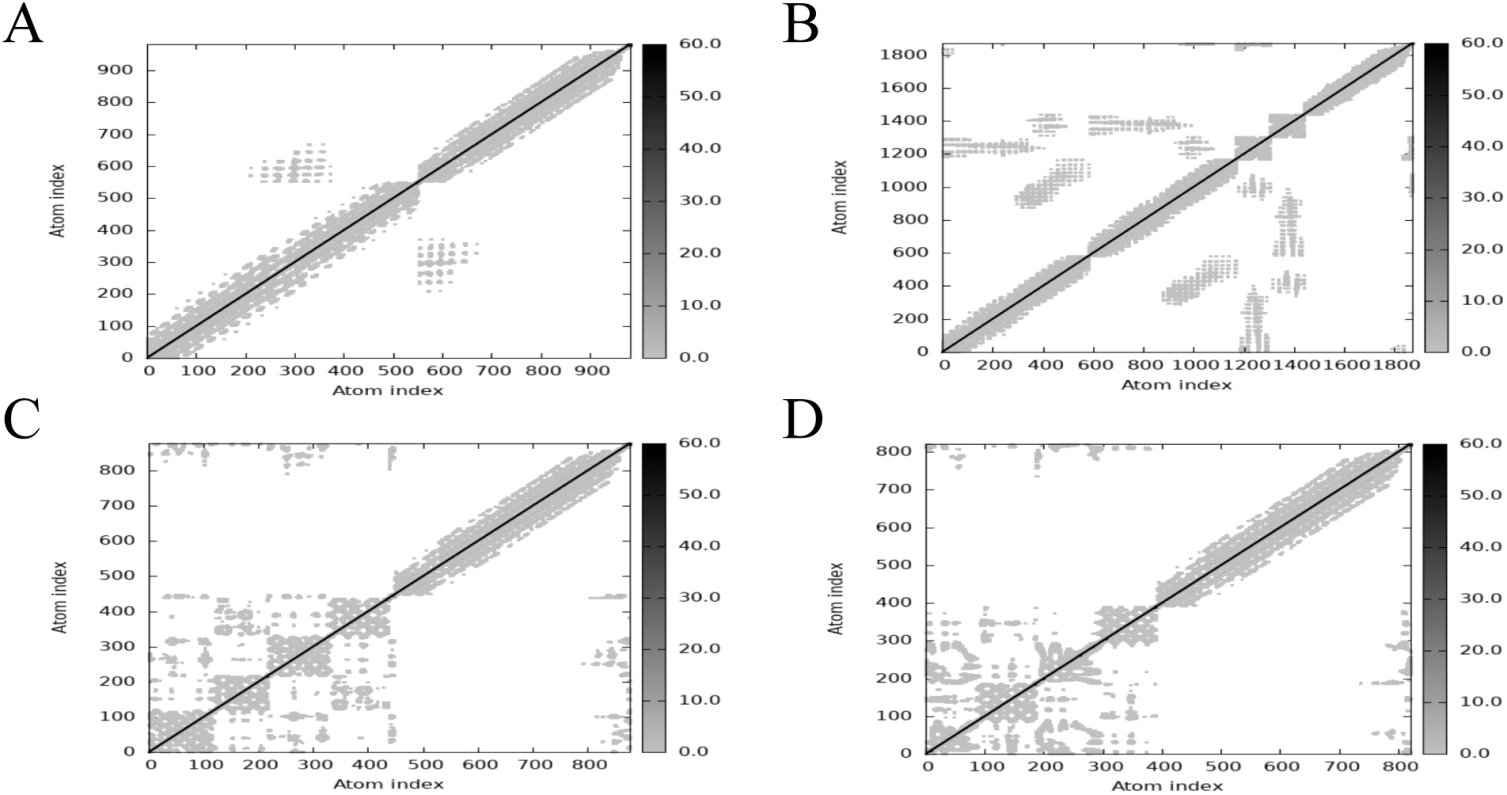
iMODS analysis revealed the Elastic network of PME-TLR2, PME-TLR4, PME-MHC I, and PME-MHC II. **(A)** The Elastic network model plot of PME-TLR2. **(B)** The Elastic network model plot of PME-TLR4. **(C)** The Elastic network model plot of PME-MHC I. **(D)** The Elastic network model plot of PME-MHC II.

The main chain deformability, B factor, variance and elastic network model of PME-TLR2, PME-TLR4, PME-MHC I and PME-MHC II were analyzed using iMODS. The results showed that most regions of the above four complexes were rigid and not prone to deformation (**Figures 5A–D**); most regions of the four complexes were less affected by thermal motion (**Figures 6A–D**); the local flexibility of the four complexes was low (**Figures 7A–D**); each complex had spring structures, indicating that the structures of the complexes were compact (**Figures 8A–D**). Based on the above results, it was indicated that PME had a high affinity and stability with TLR2, TLR4, MHC I and MHC II receptors.

### 3.6 Immunological simulation of PME

**Figure 9:**
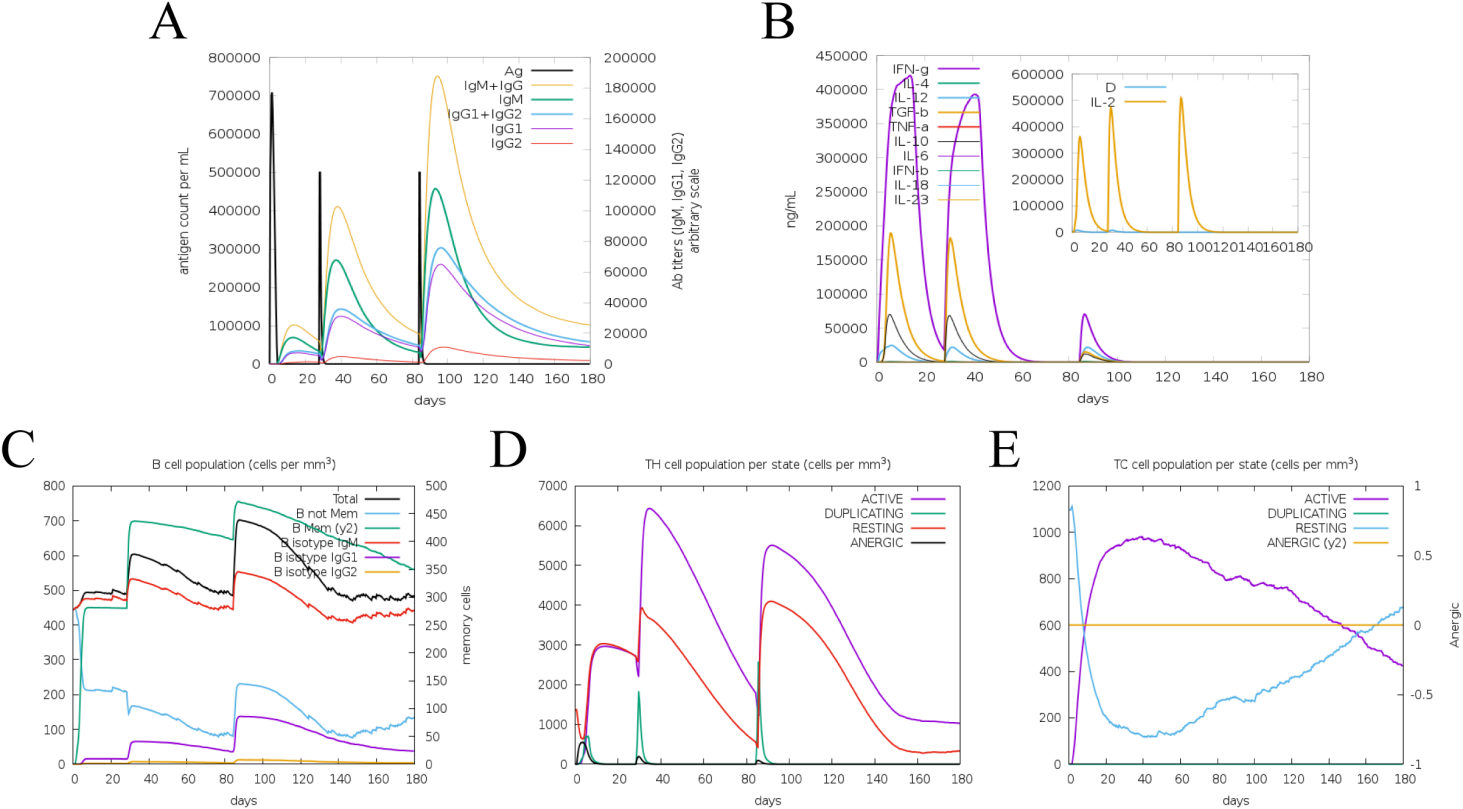
Simulate the immune response induced by PME *in vivo*. **(A)** Antibody level. Antibodies are subdivided by homotype. **(B)** Cytokine concentration. **(C)** Number of B cells. **(D)** Number of helper T lymphocytes. **(E)** Number of cytotoxic T lymphocytes.

The immune response of the multi-epitope protein PME *in vivo* was simulated using C-ImmSim. The simulation results showed that the levels of antibodies and cytokines such as IL-2 and IFN-γ significantly increased after vaccinations (**Figures 9A, B**). Meanwhile, higher levels of B-cell and T-cell populations were observed, indicating the activation of cellular and humoral immunity *in vivo* (**Figures 9C–E**). These results suggested that the multi-epitope protein PME could induce a strong immune response *in vivo*.

### 3.7 Preparation and characterization of His-PME and pcDNA3.1-PME

**Figure 10:**
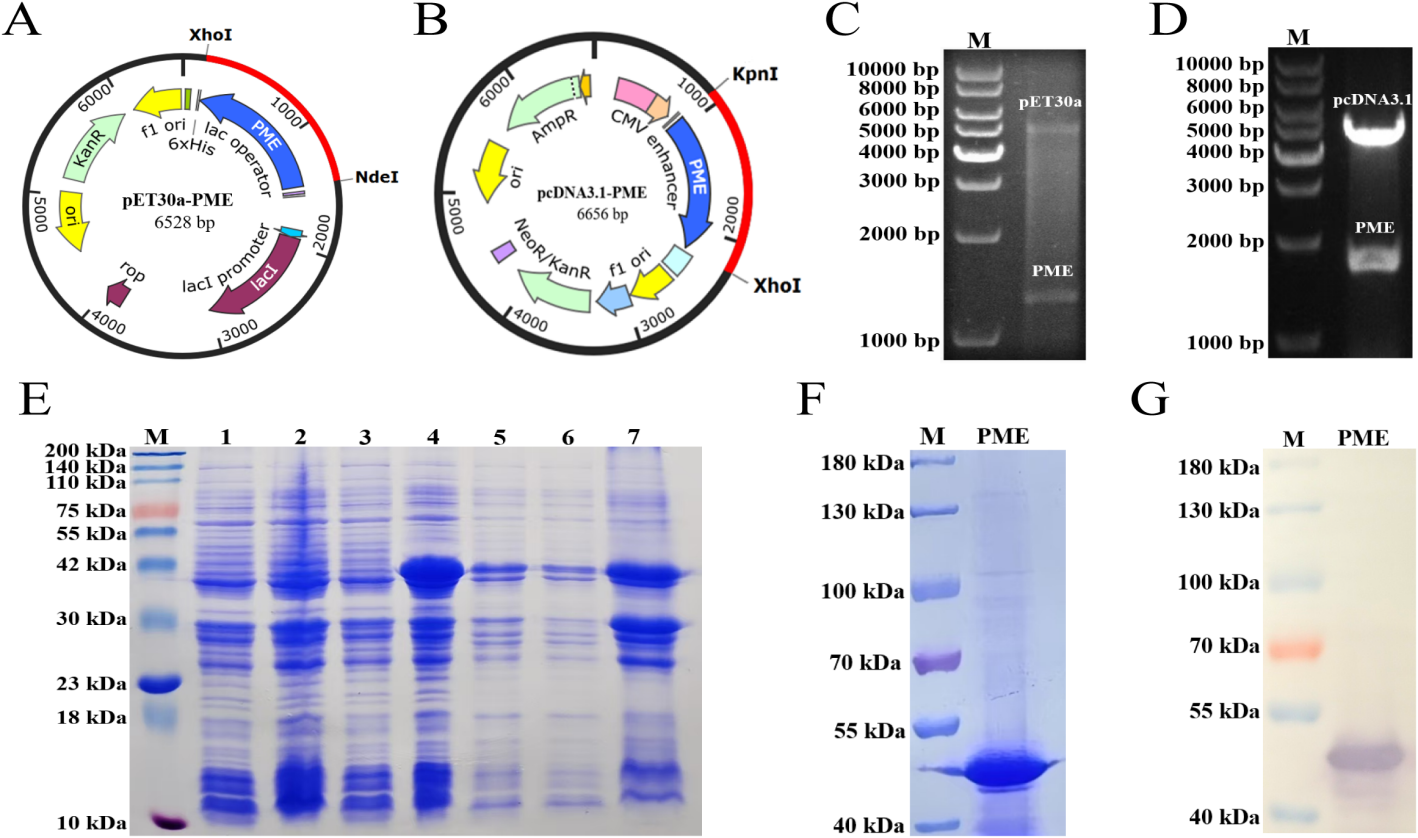
Cloning, expression and purification of His-PME. **(A)** Clone the codon optimized PME sequence (blue) into the pET30a vector. **(B)** Clone the codon optimized PME sequence (blue) into the pcDNA3.1 vector. **(C)** Identification of the recombinant plasmid pET30a-PME. The plasmid was cut with the restriction enzymes Xho I and Nde I. **(D)** Identification of the recombinant plasmid pcDNA3.1-PME. The plasmid was cut with the restriction enzymes Kpn I and Xho I. **(E)** SDS PAGE analysis of His-PME. M: 200 kDa marker. Line 1: Bacterial suspension of pET-30a BL21 without IPTG induction. Line 2: Bacterial suspension of pET-30a BL21 with IPTG induction. Line 3: Bacterial suspension of pET-30a-PME BL21 without IPTG induction. Line 4: Bacterial suspension of pET-30a-PME BL21 with IPTG induction. Line 5: Expression of His-PME protein from the whole cell lysate. Line 6: Expression of His-PME protein from the supernatant. Line 7: Expression of His-PME protein from the inclusion bodies. **(F)** The purification of the protein His-PME. M: 180 kDa marker. PME: protein His-PME after purification. **(G)** Western blot analysis of the purified protein His-PME. M: 180 kDa marker. PME: protein His-PME after purification.

The DNA sequence of PME was optimized and then inserted into prokaryotic expression vector pET30a (**Figure 10A**) and eukaryotic expression vector pcDNA3.1, respectively (**Figure 10B**). The results of double enzyme digestion indicated that the recombinant plasmids pET30a-PME (**Figure 10C**) and pcDNA3.1-PME (**Figure 10D**) were successfully constructed. SDS-PAGE showed that His-PME was successfully induced and expressed, and the protein size was consistent with the expectation (43 kDa) (**Figure 10E**). The purified protein His-PME was obtained by Ni-NTA affinity chromatography (**Figure 10F**), and the successful expression of His-PME was further confirmed by WB (**Figure 10G**).

### 3.8 Immune response of multi-epitope vaccine PME

**Figure 11:**
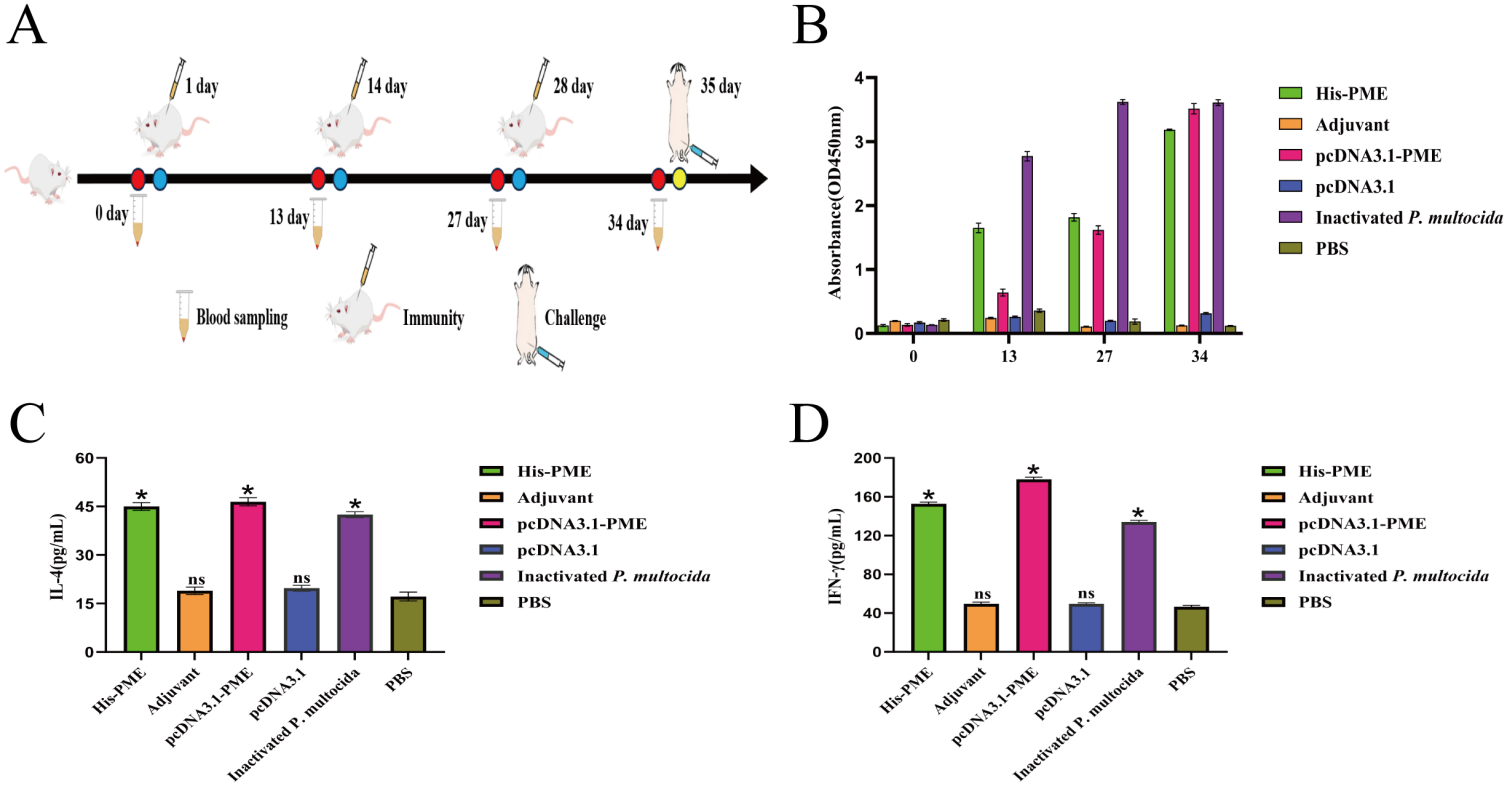
The immune response of His-PME and pcDNA3.1-PME in mice serum. **(A)** PME immunized mice protocol. **(B)** *P. multocida* serotypes D was used as the coating Antigen, and antibody levels of the mice in each groups. **(C)** The content of IL-4 in each group of immunized mice for 34 days. **(D)** The content of IFN-γ in each group of immunized mice for 34 days. The ns indicates no significant difference compared to the control group, *P < 0.05.

To evaluate whether the multi-epitope vaccine PME could induce immune response after the immunization of mice, mice were immunized three times on days 1, 14, and 28 (**Figure 11A**). The results of immune response showed that the levels of antibodies (**Figure 11B**), IL-4 (**Figure 11C**), and IFN-γ (**Figure 11D**) in inactivated *P. multocida* group, His-PME group, and pcDNA3.1-PME group were significantly higher than those in the adjuvant group, pcDNA3.1 group, and PBS group. These results indicated that multi-epitope vaccine PME could induce a strong immune response.

### 3.9 Protection against *P. multocida* in mice immunized by multi-epitope vaccine PME

**Figure 12:**
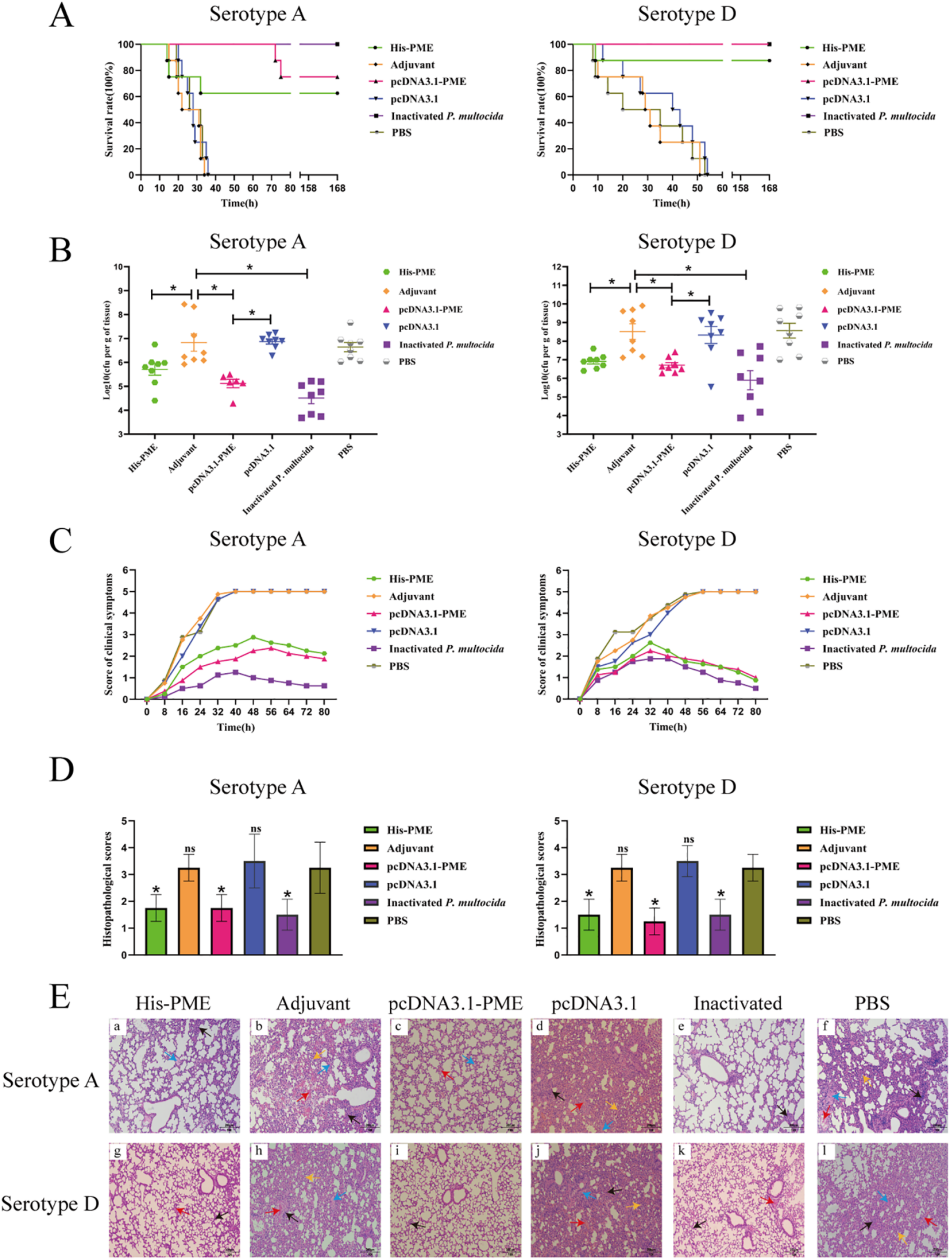
The protective effects of His-PME and pcDNA3.1-PME in immunized mice against challenges with *P. multocida* serotypes **(A, D)**. **(A)** The survival rate of mice within 7 days after challenge to *P. multocida* serotypes **(A, D)**. **(B)** Bacterial load in the lung tissue of mice after 8 hours challenge to *P. multocida* serotypes **(A, D)**. **(C)** Clinical symptom scores of mice within 80 hours after challenge to *P. multocida* serotypes **(A, D)**. **(D)** Lung histopathological scores of mice after challenge to *P. multocida* serotypes **(A, D)**. **(E)** Lung histopathological analysis of mice after challenge to *P. multocida* serotypes **(A, D)** (HE ×200). Red arrow, hemorrhage; black arrow, inflammatory cell infiltration; blue arrow, hemolysis; yellow arrow, widening of the alveolar septa. The adjuvant, pcDNA3.1 and PBS groups b, d, f, h, j and l, severe hemorrhage, inflammatory cell infiltration, hemolysis and widening of the alveolar septa. The His-PME and pcDNA3.1-PME groups a, c, g and i, mild hemorrhage, inflammatory cell infiltration and hemolysis. The inactivated groups e and k, mild hemorrhage and inflammatory cell infiltration. The ns indicates no significant difference compared to the control group, *P < 0.05.

The survival rates of mice in His-PME group, pcDNA3.1-PME group and the inactivated *P. multocida* group were 62.5%, 75% and 100% respectively after challenging with *P. multocida* serotype A, which were 87.5%, 100% and 100% respectively after challenging with *P. multocida* serotype D. Moreover, the mice in adjuvant group, PBS group and pcDNA3.1 group all died within 53 hours (**Figure 12A**).

After the mice were challenged with *P. multocida* serotype A (PM-A) and D (PM-D), the bacterial loads in the lung tissues of His-PME group, pcDNA3.1-PME group, and inactivated *P. multocida* groups (PM-A or PM-D) were significantly lower than that of adjuvant group, PBS group, and pcDNA3.1 group (**Figure 12B**). The clinical symptoms and pathological changes in the lungs of mice in His-PME group, pcDNA3.1-PME group, and inactivated *P. multocida* groups (PM-A or PM-D) were significantly alleviated and their lung histopathological scores were significantly lower than those of mice in adjuvant group, PBS group, and pcDNA3.1 group (**Figures 12C–E**). These results indicated that the immune responses and cross-immunity provided by His-PME was good, and that of pcDNA3.1-PME was even better.

## 4 Discussion

Swine pasteurellosis is a serious threat to the development of modern pig industry, and the current research on inactivated vaccine, gene deletion vaccine and subunit vaccine cannot meet the needs of the prevention and control of this disease (5). As a novel subunit vaccine with good prospect, multi-epitope vaccine can design a novel candidate antigen sequence with high immunogenicity and multi-serotype cross-protection based on B-cell epitope and T-cell epitope predicted by target antigen, and introduce spacer sequence between epitopes (57, 58). In this study, we predicted the B-cell and T-cell epitopes of six *P. multocida* antigen proteins, and connected the epitopes end to end with GSG spacer to construct the multiple epitope antigen (PME). The results indicated that PME may be an effective candidate antigen for the prevention of *P. multocida* infection in the pig industry.

At present, the relevant researches on subunit vaccines against *P. multocida* mainly focuses on Omp and other key antigens (59). Compared with single antigen immunization, the mixed immunization of these known *P. multocida* antigens can obtain better immune effect (60). Yajuan Li et al. found that the protection rates against the challenge of *P. multocida* serotype A were 33.3%, 83.33% and 83.33% respectively when vaccinating ducklings with the outer membrane proteins VacJ, PlpE and OmpH alone, and the protection rate reached 100% while vaccinating with the three proteins in combination (7). Therefore, multi-epitope vaccines obtained by combining effective epitopes of multiple key antigen proteins can theoretically effectively enhance immune efficacy.

The rapid development and wide application of bioinformatics technology have given rise to a new field in vaccine design, where vaccines based on B-cell and T-cell epitopes can induce specific immune responses (61). Currently, the research of multi-epitope subunit vaccines relies on bioinformatics analysis, which is a promising strategy (62). Compared with single epitope vaccines, *E.multilocularis* multi-epitope vaccine GILE can induce stronger immune response, effectively activating the immune system to suppress *E.multilocularis* infection (63). This technique has been applied to the development of multi-epitope vaccines of *B.melitensis* and FMDV (64, 65). In this study, we predicted 20 B-cell epitopes, 7 CTL epitopes and 11 Th-cell epitopes from six antigenic proteins (PlpE, OmpA, OmpH, VacJ, Omp87 and Cp39). Spacer sequences GSG can guarantee epitope independence without producing new epitopes, and increase the immunogenicity of the polypeptide chain (66). Therefore, we insert GSG spacer sequence between the epitopes, and obtain multi-epitope recombinant protein (PME).

The antigenicity of a protein is closely related to its secondary structure (67). In PME, the proportions of α-helices, extended chains, β-turns and random coils are 1.4%, 5.13%, 0.47% and 93.01%, respectively. The α-helix and extended chain form stable structures inside proteins, which are relatively difficult to be recognized by the immune system. β-turn and random coil are exposed to the surface of the protein and can interact better with the lymphocyte, which has a positive effect on the immunogenicity of the protein (63). The conformational rationality and model quality of multi-epitope vaccines are critical indicators for assessing whether the vaccine’s epitopes can adopt a spatial structure comparable to that of natural proteins and effectively elicit immunogenicity (68). In this study, PME demonstrates both conformational rationality and high model quality, suggesting that its spatial conformation closely resembles that of natural antigenic proteins and that it holds promise for inducing a robust immune response.

Linear B-cell epitopes are composed of contiguous amino acid residues within the primary structure of antigenic proteins. These linear sequences can be directly recognized by the variable regions of antibodies, thereby initiating an immune response. Conformational B-cell epitopes, on the other hand, consist of amino acid residues that are non-contiguous in the primary sequence but come into close proximity in the three-dimensional structure of the folded protein. This structural characteristic more closely resembles the native antigenic state and plays a crucial role in inducing antibody production (69). In this study, PME contains a substantial number of both linear and conformational B-cell epitopes, indicating its potential to elicit a broad immune response and effectively defend against pathogen invasion.

TLR2 and TLR4 are key pattern recognition receptors in the innate immune system, capable of detecting pathogens, inducing the production of pro-inflammatory cytokines such as TNF-α and IL-6, and subsequently recruiting effector cells like neutrophils and macrophages to combat microbial invasion (70, 71). MHC I molecules activate CD8(+)T cells by presenting CTL epitopes, thereby promoting the secretion of cytokines such as IFN-γ (72). MHC II molecules activate CD4(+)T cells through the binding of Th epitopes, driving Th-cell differentiation and the production of cytokines including IFN-γ and IL-4, while also supporting B cells in antibody production. In this study, PME exhibited interaction sites with TLR2, TLR4, MHC I, and MHC II, with the lowest binding energy scores observed. This suggests that PME can be recognized by these immune molecules to initiate immune responses, and that the resulting complexes demonstrate high binding affinity. The rigid regions of a complex contribute to structural stability, while moderate flexibility and thermal motion can enhance antigenicity by exposing epitopes or facilitating immune processing (73). In this study, the PME-TLR2, PME-TLR4, PME-MHC I, and PME-MHC II complexes were predominantly rigid, exhibiting a spring-like configuration with limited susceptibility to thermal motion. These findings indicate that the four complexes are relatively resistant to external perturbations, and that their flexible regions may contribute to enhancing immune activation.

In PME, the proportion of random coil is relatively higher, which helps to improve its immunogenicity. In this study, the GEL 01 RP adjuvant was used to emulsify His-PME and pcDNA3.1-PME, and then mice were immunized. His-PME and pcDNA3.1-PME immunization induced strong serum antibody levels similar to those of the inactivated vaccine, suggesting that multi-epitope vaccine PME can induce strong humoral immune response in mice. Th1 and Th2 cells play a key role in the host cellular immune response. Th1 is involved in cellular immunity and delayed inflammatory hypersensitivity and can secrete IFN-γ, while Th2 mediates humoral immune response and can secretes IL-4 (74). In this study, His-PME and pcDNA3.1-PME induced significant increases in IFN-γ and IL-4 levels, suggesting that multi-epitope vaccine PME could induce strong Th1 and Th2 responses. These results further proved that multi-epitope vaccine PME has good immunogenicity.

Currently, there are few studies on multi-epitope vaccines against *P. multocida*. The multivalent vaccine rPMT for *P. multocida* is obtained by predicting the dominant B-cell epitopes, dominant peptides and dominant T-cell epitopes of the PMT protein. After immunizing mice with rPMT, the serum antibody was significantly increased. The protection rate against the challenge with *P. multocida* serotype D was 57.1%, and the lesions of lung tissue were significantly reduced. These results indicated that rPMT could be a candidate against *P. multocida*. However, the protection provided by rPMT was limited, and it had not been confirmed whether it could provide cross-protective immunity (75). In this study, the mice immunized with His-PME and pcDNA3.1-PME had a high immune protection against PM-A and PM-D challenge, of which the CFU of lung colonized colonies was significantly reduced, and the lesions were also significantly alleviated. These results suggested that His-PME and pcDNA3.1-PME can significantly reduce the lung injury caused by *P. multocida* and provide good cross-protection against *P. multocida* infection. Taken together, PME may be a suitable candidate multi-epitope vaccine, providing a new idea for the development of vaccines against *P. multocida.*

## 5 Conclusion

In this study, multi-epitope vaccine PME was designed by predicting the B-cell and T-cell epitopes of six antigen proteins of *P. multocida*, including PlpE, OmpA, OmpH, VacJ, Omp87 and Cp39. Bioinformatics was used to analyze the physicochemical properties, secondary and tertiary structures of PME, and the results showed that PME had the advantages of strong antigenicity and high stability. In a mouse model, both protein His-PME and plasmid pcDNA3.1-PME were able to protect mice against *P. multocida* serotypes A and D. These results showed that the multi-epitope vaccine PME can provide good immune and cross-protection, and is a candidate vaccine for the prevention of *P. multocida* infection.

## Data Availability

The original contributions presented in the study are included in the article/**Supplementary Material**. Further inquiries can be directed to the corresponding author.
