# Supplementary material for: Evaluation of a multi-epitope vaccine PME for Pasteurella multocida in mouse model
Source: Front Immunol. 2025 Sep 1;16:1652907. doi: 10.3389/fimmu.2025.1652907 (PMC12433982; doi:10.3389/fimmu.2025.1652907)
Supplement: Supplementary file 1 [file Table1.docx]

| Tools | Sources (websites) |
| --- | --- |
| NCBI | https://www.ncbi.nlm.nih.gov |
| IEDB | https://www.iedb.org |
| MHCpred | https://www.ddg-pharmfac.net/mhcpred/MHCPred/ |
| Expasy ProtParam | https://web.expasy.org/protparam/ |
| ToxinPred | https://webs.iiitd.edu.in/raghava/toxinpred/design.php |
| AllerTOP v2.0 | https://www.ddg-pharmfac.net/allertop_test/ |
| VaxiJen v2.0 | https://www.ddg-pharmfac.net/vaxijen/VaxiJen/VaxiJen.html |
| SOLpro | http://scratch.proteomics.ics.uci.edu |
| DNAstar | https://www.dnastar.com/software/ |
| SignalP-6.0 | https://services.healthtech.dtu.dk/services/SignalP-6.0/ |
| DeepTMHMM | https://dtu.biolib.com/DeepTMHMM/ |
| SOPMA | https://npsa.lyon.inserm.fr/cgi-bin/npsa_automat.pl?page=/NPSA/npsa_sopma.html |
| AlphaFold 3 | https://alphafoldserver.com/welcome |
| PDBsum | https://www.ebi.ac.uk/thornton-srv/databases/pdbsum/index.html |
| ProSA-web | https://prosa.services.came.sbg.ac.at/prosa.php/ |
| Protein Data Bank | https://www.rcsb.org |
| ClusPro | https://cluspro.org/help.php |
| PyMOL | https://pymol.org |
| iMODS | https://imods.iqf.csic.es |
| C-lmmSim | https://kraken.iac.rm.cnr.it/C-IMMSIM/index.php?page=1 |
| GraphPad Prism | https://www.graphpad.com/features |

Table S1. The sources (websites) of each software.
